# Seroprevalence of Antibodies to Measles, Mumps, and Rubella among Thai Population: Evaluation of Measles/MMR Immunization Programme

**DOI:** 10.3329/jhpn.v27i1.3320

**Published:** 2009-02

**Authors:** Piyanit Tharmaphornpilas, Pornsak Yoocharean, Aim-orn Rasdjarmrearnsook, Apiradee Theamboonlers, Yong Poovorawan

**Affiliations:** ^1^ Bureau of General Communicable Diseases, Department of Disease Control, Ministry of Public Health, Nonthaburi, Thailand; ^2^ Center of Excellence in Clinical Virology, Department of Paediatrics, Faculty of Medicine, Chulalongkorn University, Bangkok, Thailand

**Keywords:** Antibodies, Immunization, Measles, Measles vaccine, Mumps, MMR vaccine, Rubella, Seroepidemiologic studies, Seroprevalence, Vaccination, Thailand

## Abstract

Stored serum specimens, from four regions of Thailand, of healthy children attending well baby clinics and of healthy people with acute illnesses visiting outpatient clinics were randomly sampled and tested for IgG antibody to measles, mumps, and rubella (MMR). The immunity patterns of rubella and mumps fitted well with the history of rubella and MMR vaccination, seroprotective rates being over 85% among those aged over seven years. A high proportion of younger children acquired the infection before the age of vaccination. MMR vaccination should preferably be given to children at an earlier age. For measles, 73% seroprotective rates among children, aged 8-14 years, who should have received two doses of measles/MMR vaccine, were lower than expected. This finding was consistent with the age-group reported in outbreaks of measles in Thailand. The apparent ineffectiveness (in relation to measles) of MMR immunization of 1^st^ grade students warrants further studies.

## INTRODUCTION

The immunization programme in Thailand, commenced in 1980, currently provides vaccines to protect against 10 childhood diseases, such as tuberculosis, hepatitis B, diphtheria, pertussis, tetanus, poliomyelitis, measles, mumps, rubella, and Japanese encephalitis, through scheduled EPI sessions in hospitals and health centres around the country. The first dose of measles vaccination was incorporated into the national immunization programme for children aged nine months in 1984. The second dose of measles vaccine was added in 1996 for 1st grade students aged seven years. In 1997, the second dose of measles vaccine was replaced by measles-mumps-rubella (MMR) vaccine. Rubella immunization was first provided to 6th grade female students aged 12 years during 1986-1998 and, later, to 1st grade students of both the sexes during 1993-1996 before being replaced by MMR vaccine in 1997 as mentioned above (1). Ages in 2004 of the population under the measles and MMR immunization programme are provided in Table 1. Surveys indicated that the coverage of 1st dose of measles vaccine was 48% in 1987, 82% in 1991, and above 90% since 1996. From the last survey, in 2003, the coverage of 1st dose of measles vaccine was 96% (2). The coverage of MMR vaccine among 1st grade students was 94% surveyed in 2004 (3).

**Table 1.** Age (in 2004) of Thai population subject to the measles, mumps and rubella immunization programmes

Immunization	Age (years) in 2004
Measles first dose∗	1-20
Measles second dose†	7-15
Mumps†	7-14
Rubella†	Female, 7-30 Male, 7-18

∗Currently for children aged 9-12 months

†Currently in MMR for children aged 7 years; MMR=Measles, mumps, and rubella

**Fig. 1.**
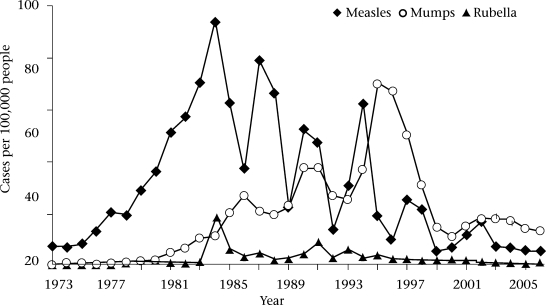
Incidence of measles, mumps, and rubella, Thailand, 1973–2006

As in other countries, the incidence of measles in Thailand has reduced dramatically since the introduction of live measles vaccine into the routine immunization programme (4-6). The number of reported measles cases reported in the National Disease Surveillance System has declined since 1984 with an outbreak peak every 3-4 years, and mortality due to measles has become extremely rare (Fig. 1). The last peak years were 2001 and 2002 (11.8-16.5 per 100,000 people) (6). The highest incidence was observed in children who were too young for vaccination (7). Outbreaks of measles in children aged less than five years occurred exclusively in hard-to-reach area where the coverage of vaccine was low. Nevertheless, outbreaks among urban and rural children aged 7-15 years still occur occasionally.

In the case of rubella, MMR vaccine is administered to school-age children aiming at preventing congenital rubella syndrome (CRS) and reducing morbidity. The incidence of rubella in Thailand is declining (Fig. 1), the reported rubella morbidity rate in 2003-2006 being only 0.61-0.78 per 100,000 people (6). No outbreak of CRS has been noted in the last 10 years but the significance of this may be questionable as Thailand does not list CRS as a notifiable disease.

The purpose of mumps vaccination in Thailand is to reduce its associated complications and morbidity. The disease-surveillance data show high outbreak peaks in 1995-1996 and, after that, the incidence declined. During 2003-2006, the incidence of mumps was 12.2-17.6 per 100,000 people (6).

Although the epidemiological changes seen in the incidence of MMR in Thailand correspond well with immunization history and levels of coverage, the national immunization programme still needs to verify actual levels of immunity. Such information would guide vaccination strategies in both preventing future outbreaks and pursuing the more ambitious targets of elimination or eradication. Accordingly, the main objective of this study was to review the seroprevalence of IgG antibodies to MMR among the Thai population after many years of vaccination against these diseases.

## MATERIALS AND METHODS

The Ethical Committee for Research in Human Subjects, Department of Disease Control, Nonthaburi, approved the study. There was no field serum specimen and evidence of data collection specifically for this study. The serum specimens were those remaining from the 2004 hepatitis immunity study (described below). Vaccination history was available only in relation to measles vaccination of children, aged less than five years, who had vaccination cards.

### Serum specimens

**Fig. 2.**
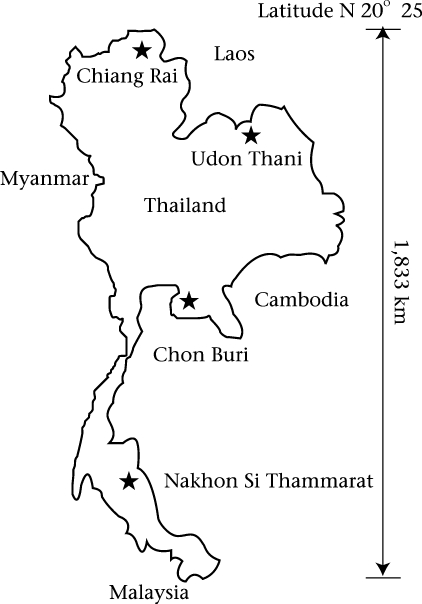
Map showing provinces from which serum samples originated

The sera used in the study were taken from 6,213 existing specimens remaining from a cross-sectional descriptive study of hepatitis immunity in the Thai population in 2004 (8,9). The samples were collected from people in four provinces: Chiang Rai, Udon Thani, Chon Buri, and Nakhon Si Thammarat, having populations broadly representative of those in the northern, northeastern, central and southern regions respectively (Fig. 2).

The participants were healthy children attending well baby clinics and healthy people with acute illnesses visiting outpatient clinics at the four provincial hospitals and two or three district hospitals in each participating province. The subjects had to meet the following criteria to include them in the study: be of good general health (apart from acute illness); have no chronic illness; not undergoing immunosuppressive therapy; and have no clinical signs of infection with HIV or other immunodeficiency disorders. Sera were randomly sampled from the serum bank to provide a separate sample for MMR. The initial sample sizes were calculated to estimate the seroprevalence among each of 0-4, 5-9, 10-14, 15-19, 20-24, 25-29, 30-39, 40-49, and 50-59 year(s) age-group for each disease. However, in analysis, the age-groups of 0-4, 5-9, and 10-14 year(s) were re-categorized to demonstrate better the seroprevelance in age-group before and after the measles and MMR vaccination age of the child.

Sera were collected and kept at −70 °C until tested. We used commercially-available ELISA IgG assays for measles (RE56611; IBL Immuno-biological Laboratories, Hamburg, Germany), mumps (RE56641; IBL), and rubella (RE57081; IBL). The laboratory results were interpreted according to the instructions of each manufacturer, except for measles IgG. The positive cut-off point for mumps was at >12 U/mL, and for rubella, at >15 IU/mL.

For measles IgG, we calibrated the test using the National Substandard of Anti-Measles-Serum, Human, 1/92 (calibrated against the 1st International Standard Anti-Measles-Serum, Human 66/202) provided by the Robert Koch Institute, Berlin, Germany. We set the positive cut-off point at 255 mIU/mL (PRN titre=120) (10,11). The calibration was intended to translate the test results into international units and to avoid the specificity of the test being too high. It indicated that the original cut-off point provided by the manufacturer was equivalent to 601 mIU/mL.

### Analysis of data

Data were analyzed by determining the geometric mean titre for each viral IgG by age-group and the seroprotective rate (which is the proportion of specimens with antibody level about the cut-off point for each viral IgG) by age-group and by province. Chi-square test (p=0.05) was used for assessing the statistical significance of the differences between proportions.

## RESULTS

### Measles IgG

In total, 1,092 serum specimens were tested for measles IgG. At a positive cut-off point of ≥255 mIU/mL, 81% of the specimens (95% confidence interval [CI] 78.8-83.5) had protective antibody levels. The seroprotective rates for those aged below 1, 1-7, 8-14, and 15-19 year(s) were 27%, 76%, 73%, and 82% respectively, which were lower than expected. For the age-group of 20 years and above, the seroprotective rates were 91-96% (Table 2). The geometric mean titre (GMT) rose sharply in those aged 1-7 year(s) compared to those aged less than one year, but there was no increase in GMT in those aged 8-14 years compared to those aged 1-7 year(s) (Table 2).

**Table 2.** Geometric mean titre of IgG antibody and seroprotective rate against measles, mumps, and rubella, by age-group

Age (years)	Measles			Mumps			Rubella		
	Sample size	GMT (95% CI) (mIU/mL)	SPR (95% CI) (%)	Sample size	GMT (95% CI) (U/mL)	SPR (95% CI) (%)	Sample size	GMT (95% CI) (IU/mL)	SPR (95% CI) (%)
0-7∗	<1 year =34	<1 year= 21.0 (8.7-50.4)	<1 year= 26.5 (13.5-44.7)	179	2.9 (1.7-5.1)	45.8 (38.4-53.4)	179	25.4 (20.7-31.0)	74.9 (67.7-80.9)
	1-7 year(s) =338	1-7 year(s)= 412.1 (334.0-508.5)	1-7 year(s)= 76.0 (71.1-80.4)						
8-14	176	367.3 (293.2-460.2)	72.7 (65.4-79.0)	157	77.9 (56.6-107.2)	89.8 (83.7-93.9)	151	64.0 (54.9-74.7)	93.4 (87.8-96.6)
15-19	105	736.2 (560.1-967.7)	81.9 (72.9-88.5)	101	48.6 (28.3-83.7)	85.2 (76.4-91.2)	103	67.7 (58.9-77.8)	95.2 (88.5-98.2)
20-24	103	962.5 (776.4-1193.2)	93.2 (86.0-97.0)	89	87.8 (55.0-140.2)	91.0 (82.6-95.8)	91	58.9 (46.3-75.0)	91.1 (82.9-95.9)
25-29	96	867.6 (691.0-1089.4)	90.6 (82.5-95.4)	94	55.8 (34.1-91.3)	86.2 (77.2-92.1)	90	48.9 (37.7-63.5)	85.6 (76.2-91.8)
30-39	81	885.2 (743.3-1054.2)	92.6 (84.0-97.0)	98	86.3 (55.2-134.7)	90.8 (82.8-95.5)	92	54.6 (42.8-69.6)	90.2 (81.8-95.2)
40-49	81	956.1 (818.2-1117.2)	96.3 (88.8-99.0)	95	120.4 (88.3-164.3)	94.7 (87.6-98.0)	97	69.6 (62.2-77.8)	94.9 (87.8-98.1)
50+	78	851.5 (690.5-1050.0)	91.0 (81.8-96.0)	98	112.4 (80.2-158.2)	94.9 (87.9-98.1)	96	65.1 (53.7-79.0)	96.9 (90.5-99.2)

∗Separated to <1 and 1-7 year(s) for measles to reflect effect of vaccination at 9 months and 7 years; CI= Confidence interval; GMT=Geometric mean titre; SPR=Seroprotective rate

Overall, the seroprotective rates of measles were similar (p=0.19) among provinces: Chiang Rai–77.4%, Udon Thani–81.3%, Chon Buri–82.3%, and Nakhon Si Thammarat–84.4%. The seroprotective rates among females were higher than among males (85% vs 76%, p<0.001). Of 139 children aged less than five years with history of measles vaccination documented in a vaccination card, 77% had protective immunity (Table 3). Analyses by age, sex, and province among this group showed no significant differences.

**Table 3.** Number and percentage of children aged less than five years with documented measles vaccination having measles protective antibody

Region	No. included in the study	No. with protective antibody	Percentage (95% CI) with protective antibody
Chiang Rai	45	39	86.7 (72.5-94.5)
Udon Thani	50	37	74.0 (59.4-84.9)
Chon Buri	21	15	71.4 (47.7-87.8)
Nakhon Si Thammarat	23	16	69.6 (47.0-85.9)
Total	139	107	77.0 (68.9-83.5)

CI= Confidence interval

### Rubella IgG

In total, 899 serum specimens were tested for rubella IgG. At a positive cut-off point of >15 IU/mL, 89% (95% CI 86.8-91.0) had protective antibody levels. The seroprotective rates were over 85% among children aged over seven years and among adults (Table 2). The GMT sharply increased at 8-14 years of age compared to the younger age-group (Table 2). Of those who were aged 20-24 years and 25-29 years, the seroprotective rates were higher among females than among males, but the differences (possibly reflecting rubella vaccination of 6^th^ grade female students during 1986-1998) were not statistically significant (96% vs 84%, p=0.14 and 89% vs 79%, p=0.34). The data indicate a higher seroprotective rate in Chiang Rai (95.1%) than in other provinces (Udon Thani–85.5%, Chon Buri–88.3%, and Nakhon Si Thammarat–87.8%, p=0.007).

### Mumps IgG

In total, 911 serum specimens were tested for mumps IgG. At the positive cut-off point of >12 U/mL in the testing manual, 82% (95% CI 78.9-84.0) had protective antibody levels. As with rubella IgG, the seroprotective rate was over 85% both among children aged seven years and above and among adults (Table 2). The GMT sharply increased at 8-14 years of age compared to the younger age-group and seemed to be higher in the older age-groups (Table 2). The differences in seroprotective rates between males and females were not statistically significant (80% vs 82%, p=0.66). The seroprotective rates were higher in Chiang Rai (89%) than in other provinces (Udon Thani–75.9%, Chon Buri–79.8% and Nakhon Si Thammarat–81.2%, p=0.001).

## DISCUSSION

Results of this study could reasonably be considered broadly representative of the Thai population but is probably not indicative of the situation of minority populations living in particular locations (e.g. the hill-tribes). The samples were collected from both urban and rural areas and from provinces in different parts of Thailand. This might be expected to introduce a bias towards urban populations having better access to healthcare but, with regard to the immunization coverage, the major differences between urban population and rural population within the same provinces would not be expected.

This study found that the immunity patterns of rubella and mumps were as would be expected from the rubella and MMR vaccination in Thailand. The effects of MMR and the previous rubella vaccination programme (from 1986 to 1998, targeting 6^th^ grade girls) was apparent in the age-group of 8-14 years and in women aged 20-30 years. The seroprotective rate of 46% for mumps and 75% of rubella among children below the age at which they would receive MMR vaccination suggests immunity acquired through continued transmission of wild virus in the community. To interrupt the transmission of virus in the future, MMR vaccine needs to be given to these younger children.

Lowering the rubella IgG antibody cut-off point to 10 IU/mL has been proposed, following epidemiologic evidence that the 10 IU/mL antibody level is protective in the vast majority of persons (12). Using this new cut-off point raised the overall seroprotective rate of rubella from 89% to 93%. It also raised the seroprotective rate among children aged 0-7 year(s) (children below the age of vaccination) from 75% to 83%, suggestive of increasingly severe transmission of wild virus among children before the vaccination age.

The higher seroprotective rates for mumps and rubella in the north compared to other regions were probably due to higher levels of virus transmission rather than a better vaccination programme. This conclusion reflects the finding that the higher seroprotective rates persisted across all age-groups, i.e. were not specific to the age-groups covered by the immunization programme. For mumps, this finding is consistent with the surveillance report showing outbreaks of mumps in the north in 1990-1991 and 1995-1996, peaking at 109.62 per 100,000 people (13,14). However, the surveillance report suggested that the incidence of rubella had been low in the north (especially in Chiang Rai where the incidence rate is 0.16-1.51 per 100,000 people) and in other regions throughout the last decade. These contradictory findings suggest possible under-reporting of rubella and, perhaps, low awareness of the importance of detection of CRS.

The level of measles antibody found in this study raised concerns. One factor which may explain the apparently low positive rate was an ‘equivocal group' having antibody levels of 150-254 mIU/mL. A measles seroepidemiology study, conducted in seven countries of Western Europe, found that the proportion of this equivocal group was high in the vaccinated age-groups, which is probably explained by lower antibody titre after vaccination (or more rapid antibody loss) than after natural infection (15). We found that 5.3% of our serum specimens fell in this equivocal group. The proportion of equivocal cases among those aged less than 1, 1-7, 8-14 and 15-19 year(s) were 2.9%, 6.5%, and 5.7% and 4.8 respectively. The 77% positive and 6% equivocal rates among children, aged less than five years, who had documented a single dose of measles vaccine at 9-12 month, were in the acceptable range.

Several hospital-based studies in Thailand found that almost 100% of infants had no antibodies to measles at nine months of age (16,17). After a single dose of measles/MMR vaccine at nine months of age, 81-91% seroconversion rates (antibody level cut-off points 320 mIU/mL in 2 studies) were obtained (16-18), which are comparable with the results of studies in other developing countries (19-21). However, the effectiveness of vaccine might vary as a result of field practice, which can be in the hands of relatively junior health officials. The effectiveness of measles vaccine (when administered at nine months of age) was evaluated during several outbreaks in Thailand, with varying results. In 1994, for example, the effectiveness of vaccine was estimated to be 35-40% in a particular population living in a remote mountainous area (22). In 1995, it was estimated at 59-70% during one outbreak (23) but 85% in another (24). In 2002, one study reported 91% (25) while another reported 87% (26).

Unexpectedly, a greater seroprotective rate or higher GMT for measles was not seen among children aged 8-14 years (supposed to have received the MMR vaccine at the age of 7 years) compared to those aged 1-7 year(s). We are unsure of the reasons for this but two hypothetical explanations seem to be plausible. The first, although not documented elsewhere, is a rapid waning of immunity after vaccination or secondary vaccine failure among school-age children. That is, immunity could have waned in children who were vaccinated but not subsequently exposed to circulating virus (27,28). The second possibility would be a potency problem with the measles component of MMR vaccine used in the programme.

While some issues remain unresolved, the results of the present study do suggest explanations for Thailand continuing to have outbreaks of measles among school and college students. During 2006-2007, there were nine outbreaks of measles reported to the National Epidemiology Office (29). Of these, five occurred among the minority populations with low vaccine coverage, i.e. hill-tribes, refugees, and displaced persons. Another four outbreaks occurred among both urban and rural populations, with outbreak sizes ranging from three to 86 patients. The populations affected were secondary school students (86 and 7 persons) in two outbreaks, vocational school students (46 persons) in one outbreak, and young adults (3 persons) in one outbreak. The high proportion of measles-susceptible school and college students means that Thailand may yet, at some point, have a nationwide outbreak. ‘Mop-up' and ‘catch-up' campaigns have reportedly been successful in raising the immune proportion of the population in many countries without major adverse effects (30,31). It may be prudent to consider similar campaigns in Thailand before an explosive outbreak occurs.

Our conclusion is that, at the time of this study, the majority of the Thai population aged over seven years was immune to mumps and rubella. MMR immunization for the 1^st^ grade students sharply reduced the number of susceptible children to mumps and rubella; however, a large proportion of younger children acquired the infection before the age of vaccination. To reduce the natural spreading of the diseases, MMR vaccination should be given to children at an earlier age. For measles, high susceptibility was found among children aged 8-14 years. This finding was consistent with the age-group reported as affected during outbreaks of measles in Thailand. Ineffectiveness towards measles of MMR immunization administered to the 1^st^ grade students warrants further study.

## ACKNOWLEDGEMENTS

This study was funded by the Ministry of Public Health, with in-kind assistance from the Center of Excellence in Clinical Virology, Chulalongkorn University, Bangkok. The authors thank field staff involved in collecting samples for the original hepatitis seroprevalence survey; the Department of Medical Science of the Ministry of Public Health, the IVD office of the World Health Organization; the Robert Koch Institute; and several colleagues who provided valued comments on earlier drafts. The work is attributed to the Ministry of Public Health, Thailand.
